# Decellularized Wharton’s Jelly from human umbilical cord as a novel 3D scaffolding material for tissue engineering applications

**DOI:** 10.1371/journal.pone.0172098

**Published:** 2017-02-21

**Authors:** Sushma Jadalannagari, Gabriel Converse, Christopher McFall, Eric Buse, Michael Filla, Maria T. Villar, Antonio Artigues, Adam J. Mellot, Jinxi Wang, Michael S. Detamore, Richard A. Hopkins, Omar S. Aljitawi

**Affiliations:** 1 Department of Bioengineering, University of Kansas, Lawrence, Kansas, United States of America; 2 Department of Hematology/Oncology, University of Kansas Medical Center, Kansas City, Kansas, United States of America; 3 Cardiac Regenerative Surgery Research Laboratories, Children’s Mercy Hospital and Clinics, Kansas City, Missouri, United States of America; 4 Department of Anatomy and Cell Biology, University of Kansas Medical Center, Kansas City, Kansas, United States of America; 5 Department of Biochemistry and Molecular Biology, University of Kansas Medical Center, Kansas City, Kansas, United States of America; 6 Department of Plastic Surgery, University of Kansas Medical Center, Kansas City, Kansas, United States of America; 7 Department of Orthopedic Surgery, University of Kansas Medical Center, Kansas City, Kansas, United States of America; 8 Department of Chemical and Petroleum Engineering, University of Kansas, Lawrence, Kansas, United States of America; Indian Institute of Toxicology Research, INDIA

## Abstract

In tissue engineering, an ideal scaffold attracts and supports cells thus providing them with the necessary mechanical support and architecture as they reconstruct new tissue *in vitro* and *in vivo*. This manuscript details a novel matrix derived from decellularized Wharton’s jelly (WJ) obtained from human umbilical cord for use as a scaffold for tissue engineering application. This decellularized Wharton’s jelly matrix (DWJM) contained 0.66 ± 0.12 μg/mg sulfated glycosaminoglycans (GAGs), and was abundant in hyaluronic acid, and completely devoid of cells. Mass spectroscopy revealed the presence of collagen types II, VI and XII, fibronectin-I, and lumican I. When seeded onto DWJM, WJ mesenchymal stem cells (WJMSCs), successfully attached to, and penetrated the porous matrix resulting in a slower rate of cell proliferation. Gene expression analysis of WJ and bone marrow (BM) MSCs cultured on DWJM demonstrated decreased expression of proliferation genes with no clear pattern of differentiation. When this matrix was implanted into a murine calvarial defect model with, green fluorescent protein (GFP) labeled osteocytes, the osteocytes were observed to migrate into the matrix as early as 24 hours. They were also identified in the matrix up to 14 days after transplantation. Together with these findings, we conclude that DWJM can be used as a 3D porous, bioactive and biocompatible scaffold for tissue engineering and regenerative medicine applications.

## 1. Introduction

Disease or trauma to the human body leads to damage and degeneration of tissues, thereby requiring their repair, replacement or regeneration. Tissue regeneration requires an optimal combination of cells, scaffolds, appropriate media and growth factors. The properties of an ideal scaffold for tissue regeneration are high porosity, biocompatibility, biodegradability and mechanical properties consistent with and suitable to the location of implant [1]. Current treatment options for tissue regeneration involve the use of autografts or allografts. Nevertheless, autografts can be difficult to obtain due to expensive and painful procedures, while allografts pose the risk of infection and immune rejection. Several types of scaffolds from natural or synthetic sources (polymers, ceramics, and composites) have been developed for tissue regeneration over the years [1]. Since, such scaffolding is associated with material-specific limitations, there is growing interest in the use of biocompatible, natural bioactives, or synthetic materials as alternatives. [2–8].

Wharton’s jelly (WJ), is a firm mucoid connective tissue surrounding umbilical cord vessels [9], possessing many unique biochemical characteristics required for a scaffold. Mesenchymal stem cells (MSCs) are derived from WJ and immersed in ground substance that is rich in collagen, hyaluronan, and also containing numerous sulfated glycosaminoglycans (GAGs) [9]. MSCs widely express the archetypal hyaluronan receptor, CD44, also expressed on osteocytes, chondrocytes, and hematopoietic marrow cells [10]. WJ is a rich source of peptide growth factors, notably insulin-like growth factor-1 (IGF-1) and to a lesser extent platelet-derived growth factor (PDGF), [11] both of which are linked to controlling cell proliferation, differentiation, synthesis and remodeling of the extracellular matrix [12].

This work is based on a hypothesis that customized cell removal procedures can effectively process WJ to produce a decellularized, bioactive Wharton’s jelly matrix (DWJM). We also postulated that DWJM would provide a 3D environment specifically well suited to support undifferentiated mesenchymal cell culture. This paper details the decellularization processes used to obtain this matrix, in addition to its characterization and analysis of its structural contents. Here, we also demonstrate that WJ and bone marrow MSCs (BMMSCs) can be seeded and cultured *in vitro* upon this matrix. We further studied the gene expression profiles of these MSCs when seeded on our 3D scaffold, and also assessed the biocompatibility of our matrix *in vivo* using a murine bone defect model.

## 2. Materials and methods

Human umbilical cord collection, WJMSCs and WJ tissue harvest followed by decellularization were performed in accordance with the approved University of Kansas Medical Center’s Institutional Review Board protocol # HSC 12129 (title—Decellularization of umbilical cord Wharton’s jelly for tissue regenerative applications including avascular necrosis) at the University of Kansas Medical Center. Consents were collected from donors by obtaining their written signatures on the approved informed consent form. Umbilical cords were immediately collected from consented mothers with full-term pregnancy after normal vaginal delivery. The umbilical cord was placed in a transport solution made of Lactated Ringer’s solution supplemented with penicillin 800 U/mL (Sigma-Aldrich, St. Louis, MO), streptomycin 9.1 mg/mL (Sigma-Aldrich), and amphotericin 0.25 mg/mL (Sigma-Aldrich) and immediately refrigerated at 4°C. The decellularization process was initiated within 72 hours of umbilical cord collection.

### 2.1 Decellularization process

The decellularization procedure has recently been described in our earlier publication [13]. Briefly, fresh human umbilical cords were transported from the delivery room in a transport solution at 4°C. Umbilical cords were dissected in a laminar flow safety cabinet, by separating the matrix into large oval pieces away from the surrounding membranes and vascular structures. They were then subjected to two cycles of osmotic shock, alternating with a hypertonic salt solution containing sodium chloride, mannitol, magnesium chloride, and potassium chloride with an osmolarity of approximately 1,275 mOsm/L, and against a hypotonic solution of 0.005% Triton X-100 in ddH_2_O centrifuged at 5,000 rpm at 4°C.

After two cycles of osmotic shock, the tissues were subjected to an anionic detergent (sodium lauryl) and, sodium succinate (Sigma L5777), then switching to a recombinant nucleic acid enzyme, (Benzonase^™^) in buffered (Tris HCl) water for 16 hours. Following this, an organic solvent extraction with 40% ethyl alcohol was performed for 10 minutes at 5,000 rpm in the centrifuge at 4°C. All of the detergent and other processing residuals were then bound and removed utilizing ion exchange beads (iwt-tmd (Sigma), XAD-16 Amberlite beads (Sigma), and Dowel Monosphere 550A UPW beads (Supelco)) in a reciprocating flow-through glass system at room temperature in ddH_2_O for 30 hours. The decellularized matrix was cryopreserved using 10% human recombinant albumin (Novozymes) and 10% DMSO (Sigma) solution in standard RPMI media, employing a material-specific computer controlled freezing profile developed to freeze at -1°C/minute to -180°C [14].

### 2.2 Isolation, expansion, and WJMSCs seeding onto DWJM

#### a. Preparation of DWJM for seeding with WJMSCs

Freshly obtained fragments of DWJM were transferred to a large petri dish and covered with phosphate buffered saline (PBS). DWJM pieces (5–7 mm in diameter) were obtained using a sterile 5–7 mm skin punch biopsy kit. The resulting DWJM pieces were cylindrical in shape and with non-uniform heights varying between 2–3 mm. DWJM scaffold volume obtained was approximately 72 mm^3^. From this point on, these pieces of DWJM will be referred to as “DWJM scaffolds”. DWJM scaffolds were transferred using sterile forceps to a large petri dish and washed twice with PBS then transferred to non-tissue culture treated plates at the time of seeding.

#### b. MSC isolation and expansion

**i. WJMSCs**—WJMSCs were isolated and expanded according to the procedures described by Wang et al [15]. Briefly, the outer layer of the cord was carefully removed and the cord was cut into smaller segments. The blood vessels were dissected from these cord segments and then cut into smaller pieces and digested with Collagenases (Worthington Biochemical Corporation, Lakewood, NJ) in low glucose Dulbecco’s Modified Eagle’s Medium (DMEM) (Sigma-Aldrich) with 10% Fetal Bovine Serum (FBS) (Atlanta Biologics, Atlanta, GA) and 1% penicillin/streptomycin (Sigma-Aldrich) overnight at 37°C to obtain WJMSCs. The WJMSCs were passaged and maintained in this low glucose DMEM-10% FBS-1% penicillin/streptomycin medium with passages 4–9 being used for the following experiments.

**ii. BMMSCs—**BMMSCs were isolated from bone marrow aspirates of healthy consented donors at University of Kansas Medical Center (HSC # 5929). The cells were isolated following standard ficoll density gradient separation method (Lymphoprep, Stem Cell Technologies, Vancouver, BC). The isolated cells were maintained in high glucose DMEM (Sigma-Aldrich), 20% FBS (Atlanta Biologics) and 1% penicillin/streptomycin (Sigma-Aldrich) at 37°C, under 5% CO_2_ and 90% humidity.

#### c. MSC characterization and phenotyping

The MACS Miltenyi Biotec MSC human phenotyping kit was used to characterize the expanded WJMSCs and BMMSCs, and analyzed by BD Flow Cytometer LSR2 (Beckton Dickinson). MSCs isolated from human umbilical cord and bone marrows were stained for CD14, CD20, CD34, CD45, CD73, CD90 and CD105.

#### d. MSC seeding onto DWJM

For each set of seeding experiments, Single-donor (n = 1) WJMSCs or BMMSCs were used. 1 x 10^6^ MSCs suspended in 50-μL culture medium were seeded on each DWJM scaffold (average seeding density 1.4 x 10^4^–4 x 10^4^/mm^3^ per DWJM scaffold) in a 48-well plate, followed by addition 1 mL of culture medium per well.

For the gene expression studies, 0.25 x 10^6^–1 x 10^6^ WJMSCs or BMMSCs from passages 4–9 were seeded on DWJM in a 24-well non tissue culture treated plate (Corning Inc., Corning, NY) for 4 to 7 days and cultured in their respective media.

### 2.3 Characterization of DWJM

#### a. DNA quantification

DNA in the samples was isolated using the Qiagen DNeasy Blood and Tissue Kit (Dusseldorf, Germany) according to the manufacturer’s instructions. Pico Green dye (Molecular Probes, Eugene, OR) was used as a label and the extracted DNA quantified fluorometrically using Quant-iT dsDNA HS (high sensitivity) Kit (Invitrogen, Carlsbad, California). The amount of extractable DNA was calculated as wet weight tissue and expressed as a percent reduction in extractable DNA relative to that for non- decellularized tissue. All analyses were run in triplicate.

#### b. Glycosaminoglycan’s (GAGs) content analysis

The Blyscan assay (Biocolor Life Sciences, UK) was used according to the manufacturer’s instructions for analysis of sulfated glycosaminoglycan content. Tissue samples from native umbilical cord WJ and DWJM were analyzed and the results were reported as μg/mg of glycosaminoglycan per wet tissue weight.

#### c. Protein identification by mass spectrometry

DWJM samples from two different umbilical cords samples were analyzed following two methods of protein extraction. For the first method, DWJM was snap-frozen using liquid nitrogen, tissue homogenized, and suspended in WJMSC culture medium as described above.

The second method used the Ready Prep Protein Extraction Kit with zwitterion detergent ASB-14 as a solubilizing agent (Bio-Rad Laboratories, Inc., Hercules, CA). This step was followed by a cleanup step to free the proteins of ionic contaminants by selective precipitation of detergents, lipids using Ready Prep 2-D Cleanup Kit (Bio-Rad Laboratories, Inc., Hercules, CA). The protein pool present in the extracts was denatured in 6M guanidine hydrochloride, reduced, alkylated, and subsequently digested for 18 h with sequencing grade trypsin (12 ng/L, Promega, Madison, WI) at 37°C. Following enzymatic digestion, the extracted peptides were concentrated to a final volume of 50 μL on a Centrivac Concentrator (Labconco, Kansas City, MO). The peptide extracts were analyzed by reversed phase chromatography using a 2D NanoLC HPLC (Eksigent Technologies, Dublin, CA) coupled to a Linear Thermo Electron Quadripole Electron Trap—Fourier Transformation (LTQ FT) mass spectrometer (Thermo Fisher Scientific, Waltham, MA). The mass spectrometer was controlled by the Xcalibur software to perform continuous mass scan on the FT in the range of 400–1900 m/z at 50,000 resolutions, followed by MS/MS scans on the ion trap of the six most intense ions. All tandem mass scans were searched using the Proteome Discoverer (version 1.3, Thermo Fisher) against a human protein database using trypsin cleavage specificity, with a maximum of 2 missed cleavages. The following variable modifications were selected: oxidation of M (methionine), deamidation of N (asparagine), and Q (glutamine), and carboxymethylation of C (cysteine) selected as fixed modifications, with a maximum of 4 modifications/peptides allowed. Estimation of false discovery rate (FDR) was conducted by searching all spectra against a decoy database. For protein identification an FDR >1% (high confidence) was defined for all peptides of interest, which were subsequently reviewed manually.

### 2.4 Evaluating seeded WJMSC adherence to and penetration of DWJM scaffolds

#### a. Confocal microscopy

To assess WJMSCs attachment to DWJM scaffolds after cell seeding and culture, scaffolds were transferred to a viewing chamber for confocal microscopy examination using a Fluoview scanning laser confocal microscope (Olympus, Center Valley, PA). Prior to viewing, the seeded DWJM scaffolds were rinsed twice with PBS and incubated with 1 mL culture medium containing 2 μg Calcein stain (Molecular Probes, Eugene, OR). Calcein is a cell-permeant dye that is converted to green-fluorescent Calcein when in live cells. Using this stain, we tracked live WJMSCs after their being seeded onto DWJM scaffolds at 2, 24 and 48-hour intervals.

#### b. Dual beam electron microscopy

MSCs were seeded on DWJM as described above and cultured in their appropriate media for 7 days. The matrix with cells was collected after 24 hours and at day 7 and was fixed overnight in 4% paraformaldehyde (VWR, Randor, PA) in PBS at 4°C. Tissue specimens were washed three times in PBS then stained with 2% osmium tetroxide (OT) for 24 hours to label lipids. Since OT gives off a strong electron backscatter signal, OT- stained samples were again washed three times for 10 minutes in PBS. All samples were gradually dehydrated with ethanol and cleared in xylene, before being embedded in paraffin. Samples were sectioned to a thickness of 10 μm or 20 μm using a microtome (Leica, Buffalo Grove, IL) and mounted on Super Frost glass slides (Thermo Fisher, Waltham, MA). OT-stained samples were deparaffinized using two 3 minute washes of xylene and critical-point dried in 100% ethanol using an Autosamdri 815B super-critical dryer (Tousimis, Rockville, MD) Samples were sputter-coated with 5 nm of copper using a Q150T Turbo-Pumped Sputter Coater (Quorum Technologies, West Sussex, and United Kingdom) and then imaged on a Versa 3D Dual Beam electron microscope (FEI, Hillsboro, OR) at a voltage of 30 kV. An Everhart-Thornley detector (ETD) and circular backscatter (CBS) detectors were used to detect secondary electrons and backscatter electrons, respectively.

#### c. Live cell imaging

DWJM scaffolds of 30μ thickness were placed in a 12 well plate and the matrix blocked with 3% BSA for 2 hours and subsequently incubated with 3 μL anti-fibronectin antibody [F1] (Alexa Fluor^®^ 488) (Abcam, Cambridge, MA) in the dark for 12–15 hours at 4°C. Immediately prior to being seeded onto DWJM scaffolds, the WJMSCs were cultured in a 12 well plate and labeled using the CellVue^®^ Burgundy Labeling Kit (Affymetrix eBioscience, Santa Clara, CA) according to the manufacturer’s instructions. Briefly, 2.5–5 x 10^5^ WJMSCs were seeded on the labeled matrix, and cultured for 24 hours at 37°C. Imaging was made on a Leica 10X HC PL Fluotar 506505 objective of a semi-automated Leica DMIRE2 inverted epifluorescent microscope outfitted with a Ludl Bioprecision motorized stage, Sutter Instruments Xenon Lamphouse with shutters and a Retiga SRV CCD camera controlled by customized TiLa KU (KU Time Lapse) acquisition and image processing software. Regions under study/interest were imaged in Bright field, GFP (Chroma 41001 HQ480/40 excitation HQ535/50 Emission) and CY5 (Chroma 49006 ET620/60 excitation, ET700/75 emission). DWJM interaction with WJMSCs was imaged at 15-minute intervals over an 18-hour period.

#### d. Scanning electron microscopy (SEM)

The DWJM scaffolds were fixed in 2% glutaraldehyde for SEM processing. The fixed samples were washed with PBS for 10 minutes, placed into buffered 1% osmium tetroxide for 1 hour, and then washed 3 times each for 10 minutes in distilled water. The samples were then dehydrated through a graded series of ethanol at concentrations 30%, 70%, 80%, 95%, and 100% for 15 minutes each. Following this, the samples were critical point dried in CO_2_ in a model EMS 850 dryer (Electron Microscopy Sciences, Hatfield, PA), then mounted onto aluminum and coated with gold in a Pelco SC-6 sputter coater. The samples were finally viewed using a Hitachi S-2700 scanning electron microscope.

#### e. Transmission electron microscopy (TEM)

Scaffolds for TEM were rinsed in a buffer prior to fixing in 1% to 2% osmium tetroxide for 1 hour. After osmication, the scaffolds were dehydrated in an ethanol series of 30%, 70%, 80%, 95%, and 100%, 10–15 minutes for each concentration, then placing them in propylene oxide (PO) twice for 10 minutes. To enhance tissue infiltration, the scaffolds were then placed in an equal mixture of resin and PO overnight. At this point, the 1:1 mixture mix was removed from the tissue and fresh 100% resin mixture added to the sample, and allowed to sit on a platform rocker for at least 30 min. The samples were subsequently covered in resin and finally were placed in 60°C oven overnight to cure the resin. The samples were sectioned afterwards using a Leica UCT ultra microtome slicing at 80 nm in thickness, contrasted with 4% uranyl acetate and Sato's Lead Citrate and viewed at 80 KV with a JOEL JEM-1400 TEM.

### 2.5 Histology and immunohistochemistry

DWJM scaffolds were fixed in either 10% formalin or 4% paraformaldehyde, embedded in paraffin, sectioned, and stained. Slides were reviewed using an Olympus BX40 microscope and pictures were acquired using a DP72 digital camera (Center Valley, PA).

#### a. Gomori’s trichrome staining

Tissue sections were stained with trichrome stain kit—Richard Allan 87020 (Thermo Fisher, Waltham, MA) according to the manufacturer’s instructions. Using this stain, the nuclei were stained bluish-black to black, cytoplasm, muscle fibers and keratin stained red, while collagen and mucus stained blue.

#### b. Immunohistochemistry

Immunohistochemistry staining was performed at room temperature using an IntelliPATH FLX^™^ automated stainer (Biocare Medical, Concord, CA) at room temperatures. Briefly, after deparaffinization, sampleswere blocked in 3% hydrogen peroxide for 10 minutes, rinsed and blocked in strepatividin/biotin (Vector Laboratories, Burlingame, CA.). The samples were again rinsed and stained for 60 minutes at room temperature with 1:200 dilution hyaluronic acid binding protein (RMD Millipore, Danvers, MA) followed by a 15 minute labeling with horse radish peroxidase (HRP) (Dako, Carpinteria, CA). Chromogenic detection of the enzyme conjugate was made using 3,3’ diaminobenzidine (DAB) (DAkp, Carpinteria, CA) when applied for 5 minutes with slides counterstained with hematoxylin.

For collagen immunohistochemistry, after deparaffinization and rehydration, the tissue sections were incubated with a primary antibody against Collagen I (Abcam, Cambridge, MA). Following a 30-minute incubation at room temperature and rinsing, the sections were incubated with secondary antibody, goat anti-rabbit HRP-polymer MACH 2 rabbit HRP-polymer (Biocare Medical, Concord, CA). Finally, collagen staining was visualized by DAB (Dako, Carpinteria, CA) and the nuclei as counterstained by hematoxylin.

Green fluorescent protein (GFP) immunohistochemistry staining was performed by incubation with primary monoclonal goat-anti GFP antibody (1:100 dilution for 45 minutes), followed by secondary MACH2 rabbit antibody (CST, Cell Signaling Technology, Danvers, MA) MACH 2 for 45 minutes on a Clinical intelliPATH FLX automated slide stainer.

### 2.6 Evaluating seeded WJMSC proliferation

The alamarBlue^®^ (AB) cell viability assay (ThermoFisher Sci, Waltham, USA) was used to assess WJMSC viability and proliferative response following seeding onto DWJM by correlating fluorescent or absorbance signal related to metabolic activity. DWJM scaffold pieces (7 mm in diameter and 2–3 mm in height) were seeded with the expanded human WJMSCs at 1 x 10^6^ cells onto each DWJM scaffold. For controls, 1 x 10^6^ cells per well were cultured as a monolayer in each well of a 24-well plate. AB was assessed at 24 and 48 hours as well as 1 week following WJMSC seeding. These experiments were performed in triplicate.

### 2.7 Evaluating WJMSC migration toward DWJM by the trans-well migration assay

WJMSC suspension at 3–7 x 10^5^ was loaded to the upper chamber of Transwell set (Costar, Corning Inc.) and the minced DWJM tissue in low glucose DMEM with 10% Fetal Bovine Serum (FBS) and 1% Penicillin/Streptomycin added to the lower chamber. After 4 hours, the trans-wells were removed and the migrated cells counted for viability using a Vi-cell (Beckman-Coulter). All the experiments were conducted in triplicate.

### 2.8 Molecular studies

#### a. RNA extraction from cells

WJMSCs and BMMSCs were cultured as a monolayer (2D) or on DWJM (3D) as described in section 2.2d for 7 days. The cells were collected at day 0 (monolayer prior seeding DWJM), day 4 and day 7 after seeding onto the matrix. WJMSCs were harvested from the scaffolds following overnight digestion with Collagenase II. The MSCs were washed twice with PBS and centrifuged at 13000 rpm, for 20 minutes at 4°C to obtain a cell pellet which was suspended in 1 mL monophasic phenol guanidine isothiocyanate (Trizol) (Life technologies) and stored at -80°C until further processing. Once all the samples were collected, RNA was extracted using the standard procedure according to the manufacturer (Life Technologies, Carlsbad, CA).

Briefly, the RNA was separated from the aqueous phase using 0.2 mL chloroform, then 0.7 volumes of isopropanol added with centrifugation to precipitate RNA. The RNA pellet was washed twice with 75% ethanol and dissolved in 30–50 μL nuclease-free water. RNA was quantified by Nano drop spectrophotometer 8000 (Thermo Fisher Scientific). First, 1.5μg of RNA was treated and isolated using the DNA-*free*^™^ DNase l kit (Life Technologies). Then a high capacity cDNA reverse transcription kit (Applied Biosystems) was used to generate cDNA from the extracted total RNA samples by a Bio-Rad T100 thermal cycler.

#### b. Quantitative real-time PCR analysis

The quantitative real-time PCR (qPCR) reactions (20 μL) were performed with the TaqMan gene expression master mix (Life Technologies), and TaqMan array 96 well plates (Applied Biosystems, Foster City, CA) using the StepOnePlus^™^ real-time PCR system (Applied Biosystems). The primers used are described in Table 1. The qPCR reactions were performed in triplicate. StepOnePlus^™^ real-time PCR system (Applied Biosystems) was used for qPCR. Glyceraldehyde 3-phosphate dehydrogenase (GAPDH) was used as an internal control to normalize the samples to obtain cycle delta threshold over background (ΔC_T_). The delta delta CT method (2^-ΔΔCT^) was used to analyze the relative gene expression levels.

**Table 1:** Real time RT-PCR TaqMan primers and their description.

Gene symbol	Detector	Gene name
GAPDH	GAPDH-Hs99999905_m1	Glyceraldehyde-3-phosphate dehydrogenase
ACAN	ACAN-Hs00153936_m1	Aggrecan
SOX9	SOX9-Hs00165814_m1	SRY (sex determining region Y)-box 9
COL2A1	COL2A1-Hs00264051_m1	Collagen, type II, alpha 1
ALPL	ALPL-Hs01029144_m1	Alkaline phosphatase, liver/bone/kidney
RUNX2	RUNX2-Hs00231692_m1	Runt-related transcription factor 2
ITGB1	ITGB1-Hs00559595_m1	Integrin, beta 1 (fibronectin receptor, beta polypeptide, antigen CD29 includes MDF2, MSK12)
CD44	CD44-Hs01075861_m1	CD44 molecule (Indian blood group)
THY1	THY1-Hs00174816_m1	Thy-1 cell surface antigen
ENG	ENG-Hs00923996_m1	Endoglin
ALCAM	ALCAM-Hs00977641_m1	Activated leukocyte cell adhesion molecule
CD14	CD14-Hs00169122_g1	CD14 molecule
MKI67	MKI67-Hs01032443_m1	Antigen identified by monoclonal antibody Ki-67
BAX	BAX-Hs00180269_m1	BCL2-associated X protein
VIM	VIM-Hs00185584_m1	Vimentin
ACTA2	ACTA2-Hs00426835_g1	Actin, alpha 2, smooth muscle, aorta
SPP1	SPP1-Hs00959010_m1	Secreted phosphoprotein 1
COL1A	COL1A1-Hs00164004_m1	Collagen, type I, alpha 1
COL4A1	COL4A1-Hs00266237_m1	Collagen, type IV, alpha 1
COL6A1	COL6A1-Hs01095585_m1	Collagen, type VI, alpha 1
DES	DES-Hs00157258_m1	Desmin
HAS2	HAS2-Hs00193435_m1	Hyaluronan synthase 2
BGN	BGN-Hs00156076_m1	Biglycan
VCAM1	VCAM1-Hs01003372_m1	Vascular cell adhesion molecule 1
NOS3	NOS3-Hs01574659_m1	Nitric oxide synthase 3 (endothelial cell)
PCNA	PCNA-Hs00427214_g1	Proliferating cell nuclear antigen

### 2.9 Animal studies

#### 2.9.1 Animal surgeries

Prior to the animal studies, DWJM scaffolds were washed twice in PBS and pre-incubated in low glucose DMEM (Sigma-Aldrich) with 10% FBS (Atlanta Biologics) and 1% penicillin/streptomycin (Sigma-Aldrich) for 24 hours at 37°C, 5% CO_2_ and 90% relative humidity. After incubation, DWJM scaffolds were washed multiple times in PBS to remove excess media before transplantation. All the animal experiments were performed in strict accordance with the approved University of Kansas Medical Center Institutional Animal Care and Use Committee (IACUC) protocol # 2013.2158. All the animal studies were performed on transgenic 10kB DMP1—Cre floxed mice (6–8 week old) expressing green fluorescent protein (GFP)[16]. Mice were anesthetized with intraperitoneal injection (IP) of ketamine (90–150 mg/kg) (Vedco) and xylazine (7.5–16 mg/kg). Buprenorphine SR (0.15–0.5 mg/kg) (Zoopharm pharmacy) was given subcutaneously immediately before the surgical procedure for analgesia. One midline skin incision of approximately 1cm in length was made on the dorsal surface of the cranium, followed by the separation of skin and periosteum. A full-thickness parietal bone defect (5.0-mm in diameter) was implemented with a trephine bur (Fine Science Tools, Foster City, CA) attached to an electric Dremell hand piece (Ideal micro drill, Harvard apparatus, Holliston, MA). The defect was left empty (n = 4) or filled with the decellularized matrix (n = 4) and the skin incision was sutured with 5–0 coated Vicryl (polygalactin 910) (Ethicon^™^, Johnson and Johnson Co., New Brunswick, NJ). Heat was provided during the entire procedure and recovery by circulating warm water blankets to protect the animals from hypothermia. Post—surgery, the animals were monitored for signs of abnormal behavior, paralysis, or infection at the surgical site such as swelling, redness and discharge, and checked daily for 3 days, followed by once every other day for the duration of the study. Animals with cranial defects, and not receiving an implant served as controls. Craniotomy defects in mice were either left un-implanted (control) or implanted with DWJM to study the cellular migration and localization by observing GFP expression. The mice were humanely sacrificed after 14 days by carbon dioxide euthanasia as the primary method of euthanasia, followed by decapitation as the secondary method in accordance with the above-mentioned institutional IACUC protocol.

#### 2.9.2 *In vivo* IVIS imaging

Mice were anaesthetized with isofluorane gas prior to imaging at 24 and 48 hours by an IVIS station (Perkin Elmer), and then euthanized according to protocol.

#### 2.9.3 Tissue samples

Cranial samples with the matrix were collected in 10% phosphate buffered formalin (Newcomen Supply) within 24 hours. Tissue specimens for the animal study were decalcified using the Rapid Bone Decalcifier solution (American MasterTech) for 5–10 minutes, paraffin embedded, sectioned vertically, and stained as described above.

### 2.10 Statistical analysis

All data were expressed as means ± standard error of mean (SEM) using a threshold of p ≤ 0.05 determined statistically significant, and analyzed by the Student’s t-test, two-way analysis of variance (ANOVA), with post-hoc Bonferroni, or non-parametric Man-Whitney U testing. A threshold of p ≤ 0.05 determined statistical significance. The statistical analyses were performed utilizing Graph Pad Prism software version 6 (Graph Pad Software, Inc.).

## 3. Results

### 3.1. Characterization of DWJM scaffold structure, biochemical components, and biomechanics

**Fig 1:**
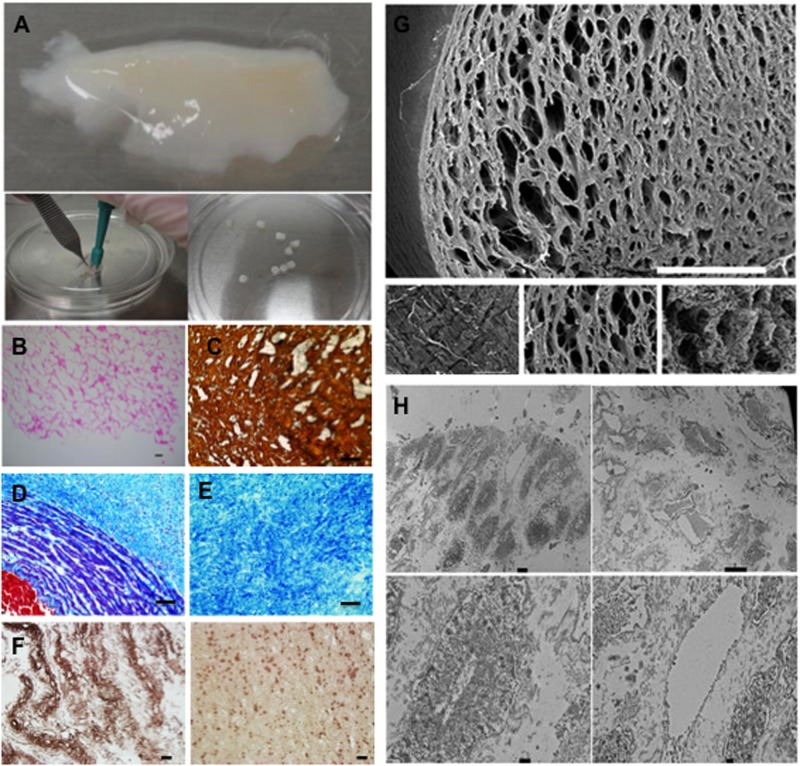
Characterization of Decellularized Wharton’s Jelly Matrix (DWJM). A) A fragment of the isolated DWJM. A skin punch biopsy kit, (right lower corner image) was used to obtain 5–7 mm DWJM scaffolds. B) hematoxylin and eosin (H&E) stained sections of the DWJM showing empty spaces. (Scale bar represents 0.1 mm.) C) Collagen I immunohistochemistry of the DWJM (scale bar is 50 μm), D) Trichrome staining images of human umbilical cord, and E) Decellularized Wharton’s jelly matrix. (Scale bar represents 50 μm.) Red color represents blood, light blue collagen, and cells/nuclei are in black/dark blue. F) Immunohistochemical staining of DWJM by anti- hyaluronic acid antibody. The matrix is rich in collagen and there is abundant hyaluronic acid expression at some parts compared to the others. (Scale bar represents 25μm.) G) Scanning electron microscopy images of DWJM. One surface appears flat with compact matrix (left lower image) while, less dense tissue with open spaces is identified in other areas (lower right and middle images). (Scale bar for the full picture is 600 μm.) H) Transmission electron microscopy images of DWJM. More electron-dense areas of DWJM (left upper image) and less electron dense areas (right upper image) are observed. No intact cells were observed in any of the panels. (Scale bar for left upper image is 2 μm, for right upper image 10 μm, and for the two lower images 500 nm.).

DWJM scaffolds were prepared from the isolated and decellularized matrix as shown in Fig 1A. Various methods were used to test the effectiveness of the decellularization process for these scaffolds. Histologically: DWJM was porous and devoid of intact cells, nuclei or other cellular components (Fig 1B). Trichrome staining of the human umbilical cord (Fig 1D) shows collagen-rich extracellular matrix in blue, nuclei/cells in dark blue/black distributed in the matrix and blood vessel wall, and blood in red. Since blood vessels are removed and the cells are lost during the decellularization process, the matrix obtained is rich in blue stain representing collagen (Fig 1E). Immunohistologically: staining for collagen (Fig 1C) and hyaluronic acid (Fig 1F) demonstrate that DWJM is rich in collagen and hyaluronic acid (Fig 1C and 1F). Scanning electron microscopy imaging: indicates that DWJM has interconnected open spaces, with sizes ranging from 20 to 100 μm (Fig 1G). Transmission electron microscopy: demonstrates an absence of intact cells the DWJM scaffold under examination (Fig 1H). Thus, the decellularization process resulted in a porous matrix, rich in collagen and hyaluronic acid and devoid of cells.

#### 3.1.1 DNA quantification studies

**Fig 2:**
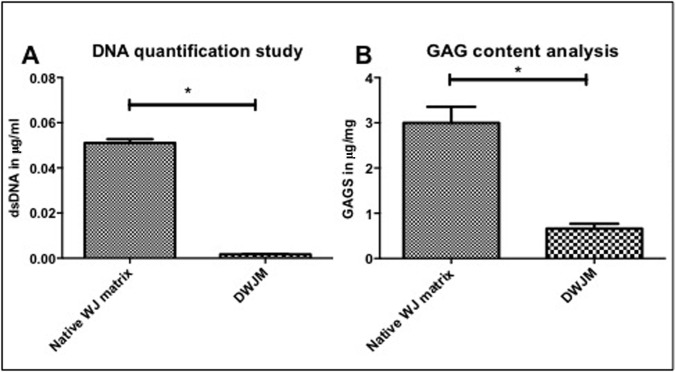
Quantification of DWJM. A) DNA quantification study performed on the matrix before decellularization and after decellularization. DWJM showed significantly less DNA compared to the native WJ matrix before decellularization. B) Glycosaminoglycan content assessment of the matrix before and after decellularization. (* Indicates statistical significance (*p* < .05)).

DNA was isolated from the native WJ matrix and from DWJM and quantified as described above. Mean dsDNA content per DWJM wet weight sample was 1.7 x 10^−3^ μg/mg (range: 1.4 x 10–3–2 x 10^−3^ μg/mg), while the mean dsDNA per WJ matrix wet weight sample was 5.1 x 10^2^ μg/mg (range: 3.17 x 10–2–7.33 x 10^−2^ μg/mg) (Fig 2A). Therefore, 96.6% ± 0.4% reduction in dsDNA was achieved for all the scaffolds analyzed, thus indicating that the majority of cells and nuclei of human umbilical cord were removed.

#### 3.1.2. Protein content analysis

Mass spectrometry revealed that the DWJM matrix pieces were composed of several structural proteins, including collagen I, III, VI, and XII. Transforming growth factor beta (TGFB) was also observed in addition to matrix proteins such as fibronectin-I, which binds to extracellular matrix components for instance collagen, heparin sulfate, tenascin and lumican. A full list of the proteins identified on mass spectrometry evaluation of DWJM is as shown in Table 2.

**Table 2:** Proteins identified in Decellularized Wharton’s Jelly Matrix (DWJM) by mass Spectrometry.

Protein name	Accession number (1)*	Sequence coverage	MW [kDa]	Theoretical. pI	Peptides number	Unique Peptides
Collagen alpha-3(VI)	219521324	13.70	278.0	8.15	18	18
Collagen type I alpha-1	110349772	6.01	138.8	5.80	7	2
Collagen type I alpha 1	180392	9.13	98.5	6.83	7	2
Collagen, type VI, alpha 1	119629727	12.06	108.5	5.43	8	8
Human Serum Albumin	55669910	16.78	65.2	5.80	7	7
Collagen type I alpha 2	825646	6.49	72.2	7.96	4	4
Collagen type VI alpha-2 isoform 2C2	115527062	13.74	108.5	6.21	8	8
Fibronectin 1	219518912	5.38	239.5	5.88	5	5
G-gamma-hemoglobin	183851	31.68	11.0	6.68	2	2
Protein kinase, DNA-activated, catalytic polypeptide	119607089	0.47	458.5	7.08	1	1
Tenascin C	156229767	4.93	210.4	4.98	4	4
TGFBI, beta-induced transforming growth factor	221044656	19.25	55.7	6.84	4	4
Lumican	4505047	15.68	38.4	6.61	3	3
Collagen, type III, alpha 1	119631314	2.66	106.3	8.10	2	2
Osteoglycin	55957237	13.06	30.4	8.34	3	3
TGFBI beta-induced transforming growth factor	37589544	3.00	75.1	7.23	1	1
Actin, alpha	119612724	12.50	30.3	5.00	2	1
Beta actin, gamma 1	194375299	10.21	37.3	5.71	2	1
HCG2044004 Human chorionic growth hormone	119628289	46.88	3.6	9.32	1	1
Collagen, type XII, alpha 1, isoform CRA_c	119569135	2.22	333.0	5.53	3	3
Hemoglobin alpha 2	13958153	59.21	8.4	7.14	2	2
Immunoglobulin heavy chain variable region	145911949	33.33	9.6	6.52	1	1
Ig G1 H Nie	229601	3.57	49.2	8.54	1	1
Decorin	119617856	14.29	28.0	8.13	2	2
Unnamed protein product	40036688	17.72	17.8	8.38	1	1
N6AMT2 Lysine N-methyl transferase	119628685	29.07	9.8	4.36	1	1
Dynein, axonemal, heavy chain 14	220732359	5.31	40.7	5.21	1	1
Chain D, Crystal Structure Of A Sparc-Collagen Complex	215261061	36.36	3.0	11.00	1	1
Golgin subfamily A member 3 (GOLGA3) protein	38174254	4.63	93.0	5.05	1	1
Glyceraldehyde 3-phosphate dehydrogenase	134254708	14.46	17.3	8.60	1	1
Triacylglycerol lipase (EC 3.1.1.3), hormone-sensitive—human	1082874	3.18	85.4	7.77	1	1
PLEKHG3 protein Pleckstrin homology domain family G	120537866	3.32	80.8	5.40	1	1
OPK V Other protein kinase group, NimA family	38502049	4.01	67.9	8.98	1	1
Plexin D1, isoform CRA_c	119599646	1.09	193.4	6.96	1	1
CDH24 Cadherin 24	28375477	10.79	26.3	5.43	1	1
Beta IV spectrin isoform sigma3	11602888	1.76	148.5	6.37	1	1
FBLN1 Fibulin-1	22761800	3.61	70.5	5.91	1	1
Unnamed protein product	40035675	3.16	68.9	9.32	1	1
Immunoglobulin heavy chain variable region	13171510	52.73	6.2	8.76	1	1
Periostin isoform thy8	166343771	3.19	80.3	8.19	1	1
Transferrin receptor protein 2	33589848	3.37	88.7	6.11	1	1
H2AFJ histone	194382012	17.12	12.1	10.40	1	1
Large tumor suppressor, homolog 2 variant	62089380	1.95	101.4	9.22	1	1
Truncated beta-globin	58201131	47.50	4.5	9.47	1	1
Dermatopontin	27151769	39.8	24	4.82	4	4
Serum albumin preproprotein [Homo sapiens]	4502027	36.29	69.3	6.28	17	17
Ig kappa chain C region	125145	32.08	11.6	5.87	2	2
Fibrinogen beta chain	399492	26.68	55.9	8.27	6	6
Fibrillin-1	311033452	17.69	312	4.93	25	25
Apolipoprotein A-I isoform X2 [Homo sapiens]	530398069	15.73	30.8	5.76	3	3
Ig gamma-1 chain C region	121039	15.5	36.1	8.19	3	3
Mimecan isoform X2 [Homo sapiens]	530391203	11.74	33.9	5.63	2	2
Fibrinogen gamma chain	20178280	9.05	51.5	5.6	2	2
Keratin, type I cytoskeletal 9	239938886	8.35	62	5.24	2	2
Fibronectin isoform 6 preproprotein [Homo sapiens]	47132549	6.8	239.5	5.88	9	9
Fibrinogen alpha chain isoform alpha pre-protein	11761629	6.52	69.7	8.06	3	3
Alpha-fetoprotein	120042	6.4	68.6	5.68	2	2
Keratin, type II cytoskeletal 1	238054406	5.59	66	8.12	3	3
Versican core protein	2506816	2.06	372.6	4.51	3	3
Fibrillin-2	238054385	1.03	314.6	4.86	2	2

* Accession number refers to the accession number in the National Center for Biotechnology Information (NCBI) protein database.

#### 3.1.3. Glycosaminoglycan content analysis

Glycoaminoglycans (GAGs) are glycoproteins with a protein core and long unbranched polysaccharide with repeating disaccharide units. Among other functions, through its carboxylic and /or sulfate ester groups, GAGs can form bridges and link collagens constructing an interconnected network of extracellular matrix to maintain and define shape of connective tissues and organs [17, 18]. Glycosaminoglycan analysis indicated that DWJM contained sulfated GAGs (mean = 0.661 ± 0.107 μg/mg), which was significantly less than that for native umbilical cord Wharton’s jelly tissue (3.0 ± 0.355 ug/mg, *<p = 0*.*05*) (Fig 2B). The loss of various cell types such as human umbilical vein cells (HUVECs), Wharton’s jelly mesenchymal stem cells (WJMSCs), and umbilical cord blood mesenchymal stem cells (UCBMSCs) during processing may account for the reduced content of GAGs (19), yet retaining some in the scaffold material.

### 3.2. DWJM scaffold seeding with WJ and BM MSCs

#### 3.2.1. MSC characterization by flow cytometry

**Fig 3:**
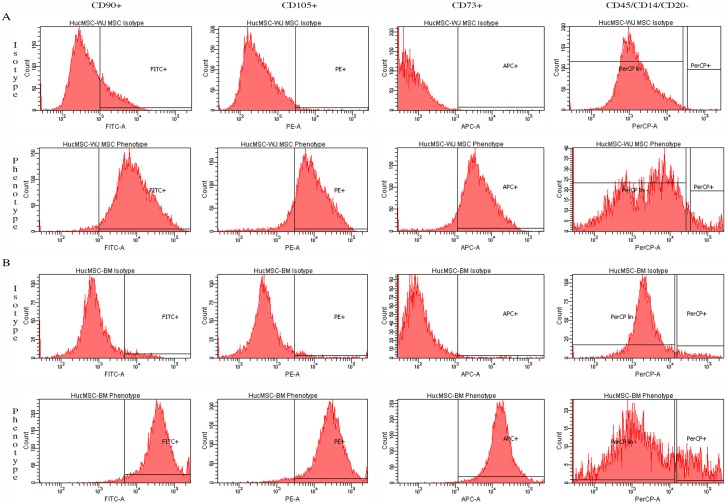
MSC characterization by flow cytometry. A) Wharton’s jelly mesenchymal stem cell (WJMSCs) and, B) bone marrow mesenchymal stem cell (BMMSCs). All MSCs stained positive for CD90 by fluoroscein isocyanate (FITC), CD105 by phycoerythrin (PE) and CD73 by allophycocyanin (APC); and they were negative for hematopoietic markers CD45, CD34, CD14 or CD11b, and CD20 as analyzed by Cell Profiler (CP) software (Broad Institute).

The isolated WJMSCs (Fig 3A) and BMMSCs (Fig 3B) were plastic- adherent and stained positive for MSC markers such as CD73, CD90, and CD105 by flow cytometry. WJMSCs and bone marrow mesenchymal stem cells (BMMSCs) were negative for hematopoietic cells markers CD45, CD34, CD14 or CD11b, CD79α or CD19**.**

#### 3.2.2 Assessment of MSC interactions with DWJM

WJMSC interactions with DWJM were studied using several modalities. Cell The adherence to and penetration into the matrix were assessed as early as 24 hours.

**Fig 4:**
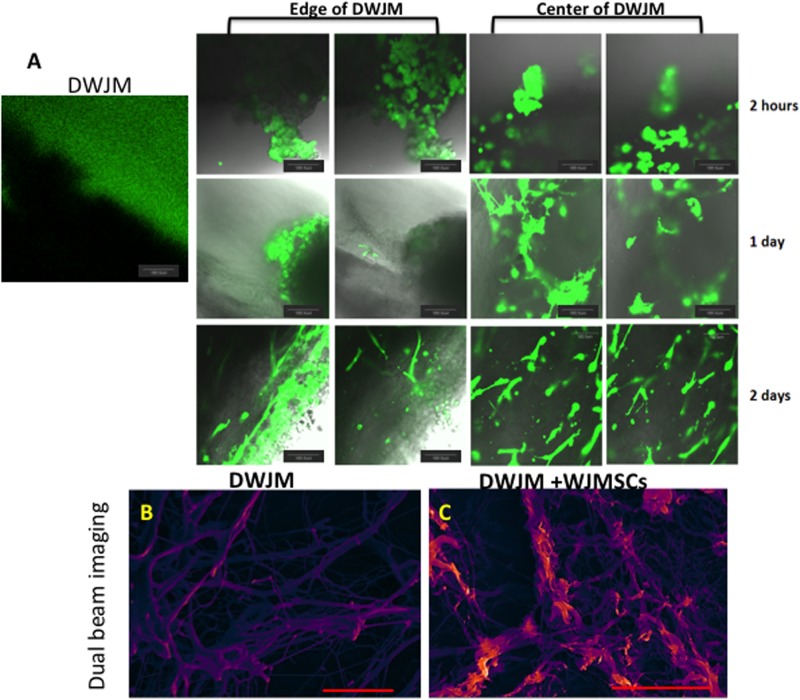
Transplantation and culturing of WJMSCs on DWJM. **A**) Confocal microscopy images of DWJM and WJMSCs on DWJM after 2 hours (upper panel), 1 day (center panel), and 2 days (lower panel) post- cell seeding. The cells are labeled with calcein acetylmethyl (AM) that stains the live cells in green. Dual beam imaging of **B)** DWJM and **C)** DWJM seeded with WJMSCs for 1 week. The Everhart-Thornley detector (ETD) is a standard secondary electron detector used in scanning electron microscopy to study topography, while the circular backscatter (CBS) is a backscatter detector that reveals lipid content when samples are stained with osmium tetroxide (OT) (red/orange). Images have been pseudo-colored to enhance definition proportional to secondary electron signal for ETD. (Scale bar is 20 μm.) DWJM appears to be a fibrous interpenetrating network with varying pore sizes, while WJMSCs were arranged along the fibers of DWJM.

WJMSC were labeled with live cell calcein green stain (CGS), and their interactions post-seeding with the DWJM scaffolds were followed by confocal microscopy. DWJM devoid of live cells did not show any fluorescence although the media in the culture well was saturated with calcein green. On the other hand, clusters of round cells were seen on the surface of seeded DWJM scaffolds within 2 hours of seeding. (Fig 4A). Elongated spindle shaped cells were noted inside DWJM within 48 hours. (Fig 4A).

Since, early interactions may not represent cell behavior at a later time point, we evaluated WJMSC interaction with DWJM at 1 week following their seeding using FEI Versa 3D dual beam imaging. DWJM fibers of varying diameters and pore sizes were stained purple (Fig 4B), while we observed spindle shaped WJMSCs arranged along the fibers of DWJM (Fig 4C).

We performed live imaging after staining the matrix with cells to further evaluate WJMSC interaction with DWJM. WJMSCs were observed migrating continuously in and out of the matrix. Cell morphology changed with the cells in the matrix becoming spindle-shaped, and developing cellular extensions while moving in and out of the matrix. Some WJMSCs could be seen in stages of division and proliferation. As the cells migrated outside the matrix, it appeared as though they were pulling part of the matrix material along with them (S1 Video). Since DWJM is a three-dimensional structure, imaging DWJM at different z-planes demonstrated that the WJMSCs were penetrating the matrix at various depths (S2 Video). WJMSCs can be seen migrating on the surface of DWJM, within DWJM, and outside of DWJM. Thus, these studies demonstrated that WJMSCs did penetrate into DWJM, continuously migrating and proliferating throughout the spaces and dimensions DWJM scaffold**.**

### 3.3 Proliferation of WJMSCs seeded onto DWJM scaffolds

**Fig 5:**
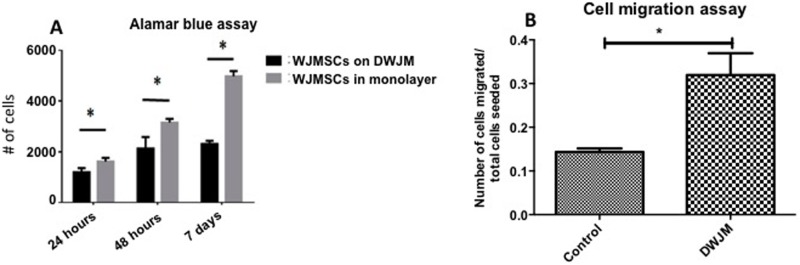
Assessing WJMSC viability and proliferation when seeded on DWJM. A) Alamar blue assay to assess the viability of cells seeded on the matrix and B) Cell migration assay performed using trans-wells with cells alone (control) and cells migrating towards DWJM, (* Indicates statistical significance *p < 0*.*05)*.

Next, we examined DWJM effects on WJMSC proliferation using the Alamar Blue cell viability assay. WJMSCs cultured as a monolayer (2D) served as controls. After 1 week, WJMSCs cultured in 3D had significantly lower fluorescence compared to WJMSCs cultured in 2D, indicating that the cell-matrix interactions allowed 3D WJMSCs to proliferate, yet at a lower extent compared to WJMSCs cultured in 2D (Fig 5A).

### 3.4 Cell migration assay

To understand early adherence of WJMSCs to DWJM, we performed an *in vitro* trans-well migration assay using DWJM scaffolds as the cell attractant. Within 4 hours, a significantly higher number of WJMSCs migrated across the trans-well when DWJM was present (Fig 5B) thereby suggesting that DWJM acts as a cell attractant. This cell attractant quality of the matrix was further investigated in our animal model.

### 3.5 Gene expression studies

A genetic approach was used to further explore the effects of DWJM on WJMSCs. Therefore; we evaluated genes responsible for cell adhesion, (to study the effects of WJMSC adhesion to DWJM), those for apoptosis, and those for proliferation (given the observed effects on WJMSC proliferation using alamar Blue). Through examination of genes associated with known types of MSC differentiation, we sought to determine the influence of DWJM on WJMSC differentiation. Similar gene expression studies were performed on BMMSCs, which served as controls.

#### A) Cell adhesion genes (Fig 6A and 6G)

**Fig 6:**
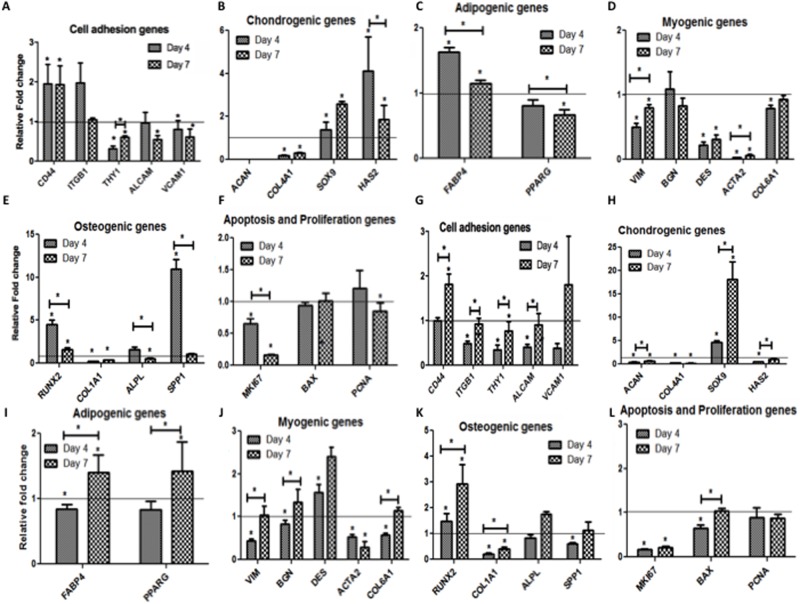
Relative fold change in the mRNA levels of the indicated genes. Panel A-F are WJMSCs on DWJM with A) Cell adhesion genes, B) Chondrogenic genes, C) Adipogenic genes, D) Myogenic genes E) Osteogenic genes, F) Apoptosis and proliferation genes. Panel G-L are BMMSCs cultured on DWJM with G) Cell adhesion genes, H) Chondrogenic genes, I) Adipogenic genes, J) Myogenic genes K) Osteogenic genes, L) Apoptosis and proliferation genes. Relative fold- change is represented on the y-axis and the genes were represented along the x-axis. The horizontal line represents the gene expression of cells before seeding at Day 0. (* Represents statistical significance p*<0*.*05*.*)*

Expression levels of cell adhesion genes CD 44, integrin subunit beta 1 (ITGB1) (CD 29), cell surface antigen (Thy1) (CD 90), activated leukocyte adhesion molecule (ALCAM) (CD 166) and vascular cell adhesion molecule-1 (VCAM1)/(CD106) were tested in WJMSCs and BMMSCs after culturing in DWJM for 7 days. There was no significant change in the expression of ITGB1, while endoglin membrane glycoprotein (ENG)/(CD105), THY1, ALCAM and VCAM1 remained below baseline levels in WJMSCS cultured in DWJM. On the other hand, after 4 days, BMMSCs cultured in DWJM showed 0.5 fold reduction in the expression of ENG, ITGB1, THY1, ALCAM and VCAM1 genes. However, by day 7 we observed an increase in expression of these genes, thus restoring their expression to baseline levels in the case of ENG, ITGB1, and ALCAM.

#### B) Chondrogenic genes (Fig 6B and 6H)

The expression of prechondrocyte and chondrocyte marker SOX9, chondrocyte markers aggrecan (ACAN), and collagen Type II alpha-1 (COL2A1) were examined in WJMSCs and BMMSCs when cultured in DWJM. ACAN and COL2A1 were undetected in WJMSCs cultured in DWJM, while ACAN was down-regulated over time in BMMSCs. SOX9 expression was up regulated in WJMSCs and BMMSCs as compared to baseline value, with BMMSCs showing a 4-fold increase at day 4 and a 15-fold increase at day 7. Hyaluronan synthase gene (HAS2) expression increased 3-fold at day 4 and 1-fold at day 7 for WJMSCs cultured in DWJM, while BMMSCs showed a decrease in HAS2 expression.

#### C) Adipogenic genes (Fig 6C and 6I)

WJMSCs and BMMSCs demonstrated expression of the adipogenic differentiation genes—fatty acid binding protein (FABP4) and Peroxisome proliferator- activation receptor– γ (PPARγ). WJMSCs cultured in DWJM demonstrated a decrease in the expression of FABP4 and PPARγ, while BMMSCs demonstrated increased expression of both genes at one week as compared to baseline value. Though these differences were statistically significant, their biological importance is unclear since the magnitude of change is small in both cases.

#### D) Myogenic genes (Fig 6D and 6J)

The expression of vimentin (VIM), byglycan (BGN), desmin (DES), actin alpha 2 (ACTA 2) and collagenase 6 (COL6A1) were studied in WJMSCs and BMMSCs cultured DWJM. At day 7 of culture, DES was down- regulated in WJMSCs, while BMMSCs demonstrated no significant change from baseline. However, the decrease in ACTA2 expression in both the cell lines was noteworthy.

#### E) Osteogenic genes (Fig 6E and 6K)

The expression of runt-related transcription factor (RUNX2), key to osteoblastic differentiation, was evaluated in WJMSCs and BMMSCs cultured in DWJM. In WJMSCs, the expression of RUNX2 increased 4-fold above a normalized baseline value of 1.0 at day 4 followed by a significant decrease at day 7. However, in the case of BMMSCs, expression of RUNX2 increased by 0.5-fold and 1.5-fold over baseline values at day 4 and 7, respectively. The expression of other osteogenic lineage markers alkaline phosphatases (ALPL) and COL1A1 were assessed over time, and it was observed that COL1A1 was down-regulated in both cell types, while there was no significant difference seen in ALPL levels in BMMSCs. WJMSCs exhibited a transient 8-fold increase in secreted phosphoprotein 1 (SPP1) expression at day 4, while WJMSCs and BMMSCs demonstrated no significant change in SPP1 expression at day 7 as compared to baseline value.

#### F) Apoptosis, proliferation, and other differentiation genes (Fig 6F and 6L)

WJMSCs and BMMSCs cultured in DWJM showed no significant change in the expression of apoptotic regulator bcl-2-like protein (BAX) at day 7, while proliferation marker MKI67 exhibited significant decrease in gene expression when compared to the respective baseline values. WJMSCs demonstrated a significant, albeit small, decrease proliferating cell nuclear antigen (PCNA) expression at week 7.

### 3.6 Animal studies

**Fig 7:**
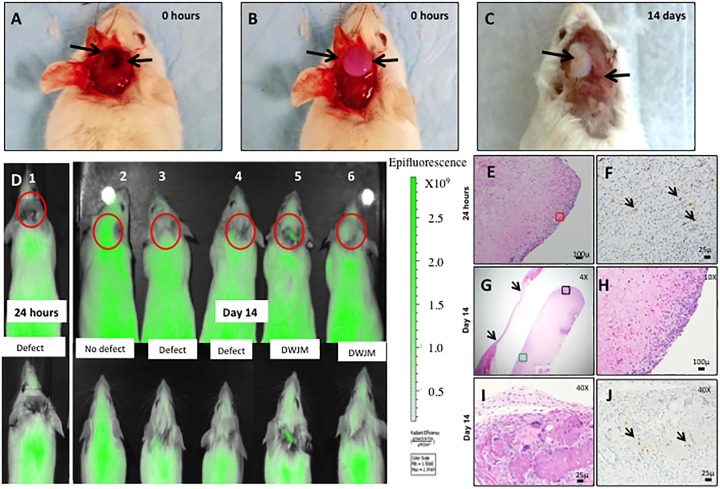
WJMSCs transplantation into an *in vivo* animal model. A) Mice with cranial defect, B) mice with cranial defect and DWJM, C) mice with cranial defect and DWJM 14 days post-surgery. Arrows in A represent the defect, B shows the DWJM and C is the defect and DWJM 14 days post-surgery. D) IVIS imaging of the mice post—surgery—1) Mice with DWJM 24 hours post- surgery; 2–6) designates mice 14 days after the surgeries. D2 is mice without any intervention, D3 and D4 are mice with the defect alone, and D5—D6 represent mice with defect and DWJM. The red circles indicate the defect sites and the inset images are a higher magnification of the defect site in mice. *The green fluorescence signal at the defect site signifies the migration of the GFP positive cells into the defect*. Images E-J represent the histology images of bone specimen with DWJM 14 days post-surgery, with image E) hematoxylin-eosin stained (H&E) section of DWJM tissue specimen 24 hours post-surgery, and image F depicts GFP immunohistochemistry staining of the same. Images G-J represent DWJM sample 14 days post-surgery with G, H and I being H&E stained sections of DWJM viewed at different magnifications as indicated in the figure. J represents the GFP immunohistochemistry of the section in image I. The arrows in image F, J represent GFP positive cells.

Since our *in vitro* studies revealed several qualities such as cell attraction and survival, although at a lower proliferation rate, we performed a set of experiments to demonstrate the ability of DWJM to attract cells *in vivo* using a murine model using GFP labeled osteocytes. Mice with defect alone (Fig 7A) were our control group, and mice with defect and DWJM served as the treatment group (Fig 7B). To study the early and late migration of GFP positive cells into DWJM, the mice were sacrificed at 24 hours and 14 days, respectively. Structural integrity of DWJM was evaluated by visual inspection after removing the skin and exposing the defect at the end of the experiment (Fig 7C). The matrix appeared intact 14 days after surgery (Fig 7C) and histological examination revealed the presence of cells (Fig 7E, 7H and 7I), some of which were also GFP positive (Fig 7F and 7J). GFP positive cells were observed in DWJM as early as 24 hours. Since these mice had GFP-labeled osteocytes, the presence of GFP positive cells by immunohistochemistry suggests that osteocytes from the neighboring bone migrated into the matrix in a diffuse pattern. Additionally, *in vivo* live animal imaging system (IVIS) was used to track GFP-labeled cells (Fig 7D) and it was observed that mice with defect alone exhibited a lack of GFP/green signal at the defect site. At 14 days, GFP signal was observed at the defect site in mice with DWJM, while there was no signal in mice with defect only. Thus, the presence of GFP signal in the area of cranial defect as evidenced by live imaging also shown by presence of GFP labeled cells focally distributed on histological sections of DWJM demonstrate that DWJM acted as a chemo-attractive and biocompatible scaffold material.

## 4. Discussion

Although a wide variety of synthetic and natural scaffolds are readily available for tissue engineering applications, natural polymers such as collagen, gelatin, silk, chitosan, and elastin pose some difficulties with processing, purity, and protein denaturation. As for synthetic materials, metal alloys are difficult to handle and are not biodegradable [2,3, 19]. Polymers such as poly lactic-acid (PLLA), polyglycolic acid (PGA), polycaprolactone (PCL), poly lactic acid-co-glycolic acid (PLGA) fit the properties of an ideal scaffold, but they are synthetic in origin and lack biological properties. Therefore, increasing needs to develop an ideal natural scaffold material [2–7, 20]. In lieu of the advantages and disadvantages of all the natural and synthetic scaffolds, we believe that DWJM has desirable features since it is a natural scaffold material that is both biocompatible and biodegradable, while promoting cellular adherence and proliferation.

Accordingly, we have demonstrated that DWJM is a biocompatible matrix that promotes WJMSC adhesion, penetration into the matrix *in vitro* while maintaining cell viability. The decellularization process adopted in our work resulted in a novel 3D matrix completely devoid of cells and dsDNA, consistent with current recommendations for tissue decellularization [21]. In our experiments, we specifically focused on the WJ matrix with total removal of vascular tissues, allantoic duct, and amniotic epithelium, in contrast to other approaches such as that proposed by Chan *et al*. [22].

Mass spectrometry analysis showed a significant residual of important extracellular matrix proteins such as collagen, fibronectin, lumican, and tenascin. These proteins play an important role in developing a scaffolding material for bone and cartilage tissue engineering applications. For example, fibronectin has been shown to enhance the quality of scaffolding material used for osteogenic differentiation [23]. Also, lumican is a matrix protein that has been correlated with *in vivo* bone formation by transplantation of *in vitro* generated osteospheroids from human mesenchymal stem cells[24]. TGF-β, identified in DWJM, also plays an important role in regulating osteogenic differentiation [25] and chondrogenic differentiation of MSCs by enhancing COL2A1 expression [26]. DWJM also contains sulfated GAGs, which are reported to enhance chondrogenic [27] and osteogenic differentiation in addition to improving cell-matrix interactions [28]. GAGs like chondroitin sulfate were also found to enhance the biological activity of collagen I scaffolds in supporting chondrocytes [29].

When WJMSCs were uniformly seeded on DWJM, cellular condensations were observed in some areas of the scaffold. This phenomenon of mesenchymal cell condensation is very similar to the process observed in early chondrogenesis resulting from cell-cell and cell-matrix interactions[30]. TGF-βI also plays a role in inducing pre-cartilage condensation [31]. Since our mass spectroscopy identified that DWJM has retained TGF-β and other matrix proteins such as collagen I, fibronectin, and tenascin,, we postulate that these proteins may have provided critical cues resulting in the WJMSCs condensation in some areas of DWJM.

The difference in timing of proliferation between WJMSCs/BMMSCs when cultured in 2D and on DWJM was consistent with an observed decrease in expression of proliferation marker Ki-67 gene, thereby indicating that MSC proliferation slows down as cells interact with DWJM scaffolds. Similar observations have been made by other researchers who also demonstrated that 3D culture conditions were found to slow down cell proliferation [32].

CD 44 is a cell adhesion receptor involved in interacting with multiple ligands such as hyaluronan, fibronectin and collagens [33]. Although BMMSCs physiologically do not express CD 44 in human or mice, Qian *et al* demonstrated that *in vitro* culture of these MSCs could result in CD 44 expression. [34–36]. In our study, we observed increased expression of CD 44 gene in WJMSCs and BMMSCs when cultured in DWJM for 7 days. Since the decellularization process adopted in this work abundantly retained hyaluronic acid in DWJM, the induction of CD 44 in both the MSCs cultured on DWJM could possibly be associated with anchoring of the cells to hyaluronic acid in the matrix. Cell surface markers such as Thy1, endoglin, ALCAM, CD 44 and VCAM have been used to isolate homogeneous MSC populations [34,37, 38]. When BMMSCs were cultured over DWJM, no significant changes in gene expression were noticed for cell adhesion molecules Thy 1 and ALCAM, while WJMSCs showed decreased expression of these genes. These subtle differences in the expression of the adhesion genes between WJMSCs and BMMSCs could be attributed to WJMSCs being native to the Wharton’s jelly matrix, while BMMSCs were introduced into a new environment.

Our gene expression studies show no clear differentiation pattern for WJMSCs when cultured in DWJM. Though RUNX2 expression is increased in BMMSCs cultured in DWJM, which is a marker of in MSC commitment to osteogenic differentiation [39, 40], SOX9 expression was also increased, which is an inhibitor of RUNX2, thus blocking osteoblastic maturation; which typically occurs during chondroprogenitor fate determination [41]. In an attempt to understand SOX9 and RUNX2 roles in osteogenesis in MSCs, Loebel *et al*. has shown that the RUNX2/SOX9 ratio can be used to screen for osteogenesis during *in vitro* osteogenic differentiation of MSCs [42]. In our experiments, the ratio of RUNX2/SOX9 showed the same decreasing trend for WJMSCs and BMMSCs cultured over DWJM (WJMSCs: 3.26 at day 4 to 0.62 at day 7; BMMSCs: 0.34 at day 4 to 0.16 at day 7) thereby indicating non-commitment to osteogenic lineage in either case. Several researchers have already demonstrated the potential of WJMSCs differentiation into myogenic lineage both *in vitro* and *in vivo* [43, 44]. Biglycan **(**BGN), a critical protein for collagen fibril assembly and muscle regeneration and alpha smooth muscle actin 2 (ACTA 2), a protein essential for maintaining cell motility, structure and integrity were significantly down-regulated in both types of MSCs. Accordingly, no lineage specific differentiation towards osteogenic, chondrogenic, myogenic or adipogenic genes was observed.

Thus, we demonstrate that DWJM is a biocompatible matrix with cell attraction properties. This is further confirmed in our animal experiments, where we have demonstrated GFP labeled osteocytes migration into the matrix as early as 24 hours by immunohistochemistry, and at 2 weeks by *in vivo* live imaging. In addition to biocompatibility, DWJM has favorable surgical characteristics like porosity, elasticity, and compressibility, which make it easy to configure in irregular or curved shapes necessary for scaffold structure. Thus, based on all these characteristics, we envision that DWJM scaffolds will have several potential applications related to tissue engineering.

## 5. Conclusions

In this manuscript we have successfully isolated, decellularized, and fully characterized the human WJ matrix. We have shown that this naturally obtained matrix can be made completely devoid of cells, yet still comprised of glycosaminoglycans especially rich in hyaluronic acid and several other key extracellular matrix proteins. We also have demonstrated that DWJM is a biocompatible matrix that allows for cellular adherence, penetration, growth and proliferation with suitable/acceptable mechanical properties *in vitro* and *in vivo*. In sum, this paper presents DWJM as a novel and natural 3D scaffold that can be used for tissue engineering and regenerative medicine applications.

## Supporting information

S1 Video Time-lapse imaging of WJMSCs seeded on DWJM. Time-lapse imaging of WJMSCs labeled in burgundy and DWJM labeled in green color (WJMSCs on DWJM are in yellow). Panel on the top left represents bright field image of WJMSCs on DWJM, top right represents labeled WJMSCs, bottom left are labeled DWJM, and bottom right shows labeled WJMSCs on DWJM. WJMSCs can be seen proliferating and migrating inside and outside the DWJM. Imaging was performed over 18 hours. (Scale bars represent 100 μm).
(AVI)

S2 Video Time-lapse imaging of WJMSCs on DWJM in different Z planes. The three panels represent WJMSCs at three different depths in the matrix as Z − 1, Z 0 and Z + 1. Scale bar = 100 μm. The movies on the top panel are labeled WJMSCs on labeled DWJM, while the videos on the bottom panel are bright field images of WJMSC on DWJM. The full Z volume for the acquisitions was 225μ through 7 steps of 37.5μ per Z-step/plane.
(AVI)
