# Correction: Decellularized Wharton's Jelly from human umbilical cord as a novel 3D scaffolding material for tissue engineering applications

**DOI:** 10.1371/journal.pone.0173827

**Published:** 2017-03-07

**Authors:** The PLOS ONE Staff

The Competing Interest Statement for this paper is incorrect. The correct Statement is: The authors have declared that no competing interests exist. The publisher apologizes for the error.
